# Prevalence and factors affecting home blood pressure documentation in routine clinical care: a retrospective study

**DOI:** 10.1186/1472-6963-10-139

**Published:** 2010-05-27

**Authors:** Michael H Kramer, Eugene Breydo, Maria Shubina, Kelly Babcock, Jonathan S Einbinder, Alexander Turchin

**Affiliations:** 1 Department of Computer Science, Harvard College, Cambridge, MA, USA; 2 Clinical Informatics Research and Development, Partners HealthCare System, Boston, MA, USA; 3 Harvard Medical School, Boston, MA, USA; 4 Center for Clinical Investigation, Brigham and Women's Hospital, Boston, MA, USA; 5 Bouvé College of Health Sciences, Northeastern University, Boston, MA; 6 Division of General Internal Medicine, Brigham and Women's Hospital, Boston, MA, USA; 7 Division of Endocrinology, Brigham and Women's Hospital, Boston, MA, USA

## Abstract

**Background:**

Home blood pressure (BP) is closely linked to patient outcomes. However, the prevalence of its documentation has not been examined. The objective of this study was to analyze the prevalence and factors affecting documentation of home BP in routine clinical care.

**Methods:**

A retrospective study of 142,973 encounters of 9,840 hypertensive patients with diabetes from 2000 to 2005 was performed. The prevalence of recorded home BP and the factors associated with its documentation were analyzed. We assessed validity of home BP information by comparing the difference between home and office BP to previously published prospective studies.

**Results:**

Home BP was documented in narrative notes for 2.08% of encounters where any blood pressure was recorded and negligibly in structured data (EMR flowsheets). Systolic and diastolic home BP in narrative notes were lower than office BP readings by 9.6 and 2.5 mm Hg, respectively (p < 0.0001 for both), consistent with prospective data. Probability of home BP documentation increased by 23.0% for each 10 mm Hg of office systolic BP (p < 0.0001), by 6.2% for each $10,000 in median income of zip code (p = 0.0055), and by 17.7% for each decade in the patient's age (p < 0.0001).

**Conclusions:**

Home BP readings provide a valid representation of the patient's condition, yet are seldom documented despite their potential utility in both patient care and research. Strong association between higher patient income and home BP documentation suggests that the cost of the monitors may be a limiting factor; reimbursement of home BP monitoring expenses should be pursued.

## Background

Home blood pressure measurements provide valuable clinical information in the treatment of hypertension[1-3]. They offer a longitudinal perspective that complements the information supplied by casual office measurements, and their importance is increasingly recognized by clinical guidelines[4-6]. Multiple studies have shown that home blood pressure correlates with clinical outcomes including strokes, left ventricular hypertrophy, renal and retinal complications and cardiovascular mortality better than office blood pressure measurements[2,7-11]. Furthermore, it has been demonstrated that models of both cardiovascular and all-cause mortality risk are improved with the addition of home blood pressure readings to office and ambulatory measurements[12,13]. Finally, home blood pressure measurements can help differentiate white coat hypertension from persistently elevated blood pressure[5].

Based on these data, many physicians encourage their hypertensive patients to monitor their blood pressure at home and use home blood pressure information during the treatment of patients[14-16]. Surveys show that this advice is widely taken up by patients, and approximately 70% of hypertensive patients monitor their blood pressures at home[16,18,19]. Despite the clear utility and wide prevalence of home blood pressure monitoring, its documentation in routine clinical care remains poorly investigated. We therefore undertook this study to analyze documentation of home blood pressure in routine clinical care.

## Methods

### Design

#### Evaluation of software classifications of the source of blood pressure readings

Sensitivity, specificity and positive predictive value of classification of blood pressure readings in narrative provider notes by the software as "home" vs. "office" (i.e. measured during the provider-patient encounter) were evaluated by comparison of the software results with manual review.

#### Documentation of blood pressure information in electronic medical records (EMR)

We carried out a retrospective analysis of EMR data to identify the source of the blood pressure reading (home vs. office) and the location of the record in the EMR (structured flowsheets vs. narrative notes). A single encounter documented in either narrative or structured data served as the **unit of analysis**.

#### Comparison of magnitude of blood pressure readings taken at home and in the office

We carried out a retrospective analysis of EMR data to compare the magnitude of patient's home blood pressure readings to the office blood pressure readings found in structured flowsheets and in narrative text. An encounter for which both a home blood pressure and an office blood pressure of the specified type (structured or narrative) were recorded served as the **unit of analysis**. Blood pressure level (systolic and diastolic blood pressures were analyzed separately) served as the **primary outcome variable**. Blood pressure reading type (office recorded in structured data, office recorded in narrative notes, home recorded in narrative notes) served as the **independent variable.**

#### Factors that affect documentation of home blood pressure in narrative notes

It is not known how patient characteristics affect patterns of home blood pressure documentation in the EMR. We analyzed the relationship between the presence of a home blood pressure reading (binary **primary outcome variable**) and the following **predictor variables**: 1) patient's office systolic blood pressure during the encounter; 2) patient's office diastolic blood pressure during the encounter; 3) patient's income as represented by the median income of the patient's zip code; 4) patient's age; 5) patient's gender; 6) patient's ethnicity; 7) patient's primary language; and 8) patient's insurance. A single encounter served as the **unit of analysis.**

### Data Sources

Partners HealthCare System is an integrated healthcare delivery network comprised of several academic and community hospitals and private physician groups in eastern Massachusetts, including the founding members Brigham and Women's Hospital and Massachusetts General Hospital. Most physicians affiliated with these two hospitals use an internally developed outpatient electronic medical record (EMR) system Longitudinal Medical Record (LMR)[20]. LMR allows for entry of both structured dictionary-based data (e.g. medications, allergies, problems) as well as narrative text (e.g. progress notes, radiology and pathology reports, and others). For the purpose of this study we compared blood pressure information obtained from the structured entries in the flowsheet section and through computational analysis of the text of narrative physician notes in the LMR. Median incomes for each zip code were obtained from the 2000 US census data. All data were de-identified.

### Patients

We included in our study all patients who were followed in primary care practices at either Brigham and Women's Hospital or Massachusetts General Hospital for at least two years between 01/01/2000 and 08/31/2005, were at least 18 years old, and had a documented diagnosis of diabetes mellitus. Patients who did not have any office blood pressures recorded during the study, did not have a zip code listed, had an invalid zip code, or were from a zip code without median income data in the US census were excluded.

### Algorithm

The program used to extract the data from narrative notes employed a semantic algorithm to identify two sets of concepts: blood pressure values and type of blood pressure reading. Possible types of blood pressure readings as classified by the program were readings taken at home, readings taken during the encounter of record (henceforth referred to as "office" BP readings), and readings taken neither at home nor during the encounter. The program utilized as a foundation a previously validated algorithm used to identify blood pressure values in narrative notes[21]. When a blood pressure value was identified, the sentence containing the blood pressure value was evaluated using empirically derived heuristics to determine whether the blood pressure was taken during the encounter or at home. If none of the heuristics applied, the blood pressure reading was classified as taken during the encounter by default. After each individual blood pressure reading in a note was classified, all of the blood pressures in the note were examined together to select the blood pressure reading(s) most likely to have been taken during the encounter. During this process, if there was at least one blood pressure reading in the note that was classified as taken during the encounter by one of the heuristics, then any blood pressure which had previously been classified as taken during the encounter by default had its classification changed to neither during the encounter nor at home.

### Study Measurements

We evaluated the accuracy of identification of blood pressure readings taken at home or in the office on a dataset of 300 narrative physician notes. Each note was manually analyzed by two trained senior pharmacy students who did not participate in the development of the algorithm. The reviewers abstracted all blood pressure readings and assigned to each of them a categorization of "home", "office" (i.e. taken during the encounter of record) or "neither" (e.g. taken during a previous provider-patient encounter). Blood pressure readings for which the reviewers did not reach an agreement were re-analyzed to establish the consensus designation. The software output was compared to the consensus rating to determine sensitivity, specificity and positive predictive value (precision) for categorization of blood pressure readings.

Home blood pressure readings documented in the structured flowsheets in the EMR were identified using manually entered notes in the Comment field (e.g. "at home", "home cuff", "average home readings", etc.). Home blood pressure readings documented in the narrative notes were abstracted using the algorithm described above. Physician notes and EMR records that had the same *service date *were treated as the same encounter for the purpose of data analysis. *Service date *in our institution's EMR indicates the date of the provider-patient encounter to which the electronic transaction pertains and may be different from the date when the record was actually modified. Home blood pressure measurements reported in provider notes were compared to office blood pressure measurements made on the day of the encounter that the note documented.

For the analysis of the data collected from narrative notes, the minimum and maximum of any blood pressures represented as a range in the narrative notes were averaged to give a single blood pressure value. Then, for each encounter, the lowest home, office, and structured blood pressure readings were selected based on the mean arterial blood pressure (MAP, defined as diastolic BP + 1/3*(systolic BP - diastolic BP)). If two blood pressure readings had the same MAP, the one with the lower systolic BP was selected as the lowest BP of the given type for the given encounter.

For the multivariable analysis of the factors that may affect documentation of home blood pressure, the office blood pressure (structured or narrative) with the lowest MAP was used to establish the predictor variables of office systolic and diastolic blood pressure. For encounters that did not have an office blood pressure documented (301 encounters), systolic and diastolic blood pressures were imputed based on the patient's office blood pressures recorded during other encounters, home blood pressure readings and all other covariates used in the construction of the multivariable model using SAS multiple imputation procedures PROC MI and PROC MIANALYZE.

### Statistical Analysis

Summary statistics were constructed by using frequencies and proportions for categorical data and by using means and standard deviations for continuous variables. Wilcoxon signed rank test was used for univariate analysis of the difference in magnitude between home and office blood pressure readings.

To analyze the relative magnitude of home and office blood pressure readings documented in structured and narrative data, we constructed a hierarchical (multilevel) mixed multivariate linear regression model with random effects to account for clustering within patients. The model adjusted for the patients' age, gender, ethnicity, primary language, insurance status and income level.

To analyze the factors that were associated with documentation of home blood pressure in narrative provider notes we constructed a hierarchical (multilevel) multiple logistic regression model. We used the GLIMMIX procedure to adjust for clustering within treating physicians and patients[22,23]. The results were combined across multiply imputed datasets using SAS MIANALYZE procedure[24,25]. We imputed missing office SBP values using regression method with the covariates planned to be used in modeling of the probability of home BP documentation and home BP values. We subsequently imputed office DBP using predictive mean matching method[26] with imputed office SBP added as a covariate to the set of independent predictors from imputation of office SBP. We generated 5 datasets, which is usually considered sufficient for a valid inference. We then constructed five multivariable logistic mixed models for the probability of home BP documentation with random intercepts for providers and compound symmetry correlation structure within patients to account for possible dependence (clustering) of observations using proc GLIMMIX, and the results were combined as proper by applying proc MIANALYZE. The model adjusted for patient's age, income level, office systolic blood pressure, office diastolic blood pressure, gender, insurance, primary language, and ethnicity. Association significance thresholds were calculated using Simes-Hochberg for multiple testing[27,28]. SAS version 9.1.3 (SAS Institute Inc., Cary, NC) was used for all analyses.

### Institutional Review Board

Partners HealthCare System institutional review board granted expedited approval of this study and waived the need for informed consent.

## Results

### Identification of Home Blood Pressure in the Text of the Notes

The reviewers identified 675 blood pressure readings in the evaluation notes. Of these, 624 BP readings were identified by both reviewers. The reviewers agreed on 558 (89.4%) of the ratings of whether the blood pressure was taken at home (kappa = 0.666) and on 601 (96.3%) of the ratings of whether the blood pressure was taken in the office (kappa = 0.924) during the encounter.

The software found 632 blood pressure readings in the same set of notes. Of the 675 blood pressure readings found by the reviewers and 632 blood pressure readings found by the software, 598 matched. These 598 instances of blood pressures were then used to evaluate the classifications of blood pressures as taken at home, taken at the office during the encounter of record, or neither as automatically generated by the software.

The specificity, sensitivity, and positive predictive value for the software classifications of blood pressure readings as home or taken in the office during the encounter of record ranged from 83.7% to 95.5% (Table 1).

**Table 1:** Accuracy of Identification of Home and Office Blood Pressure in Narrative Notes

	Sensitivity (95% CI)	Specificity (95% CI)	Positive Predictive Value (95% CI)
Measured in office during encounter of record	90.4%	88.3%	92.1%
	(88.0 - 92.8%)	(85.6 - 90.9%)	(89.8 - 94.3%)

Measured at home	83.7%	95.5%	83.7%
	(80.7 - 86.8%)	(93.8 - 97.3%)	(80.7 - 86.8%)

### Documentation of Home Blood Pressure in Electronic Medical Records

We identified 11,012 patients with a documented diagnosis of diabetes who were followed in a primary care clinic during the study period (Table 2). We have excluded 5 patients because they did not have any recorded office blood pressure measurements, 214 patients because median household income information was not available for their zip code and 953 patients because all of their notes were from providers who did not include any home blood pressure measurements for any of their patients' notes in our dataset, precluding their use in the multivariable analysis performed in this study. These patients had blood pressure documented in the electronic medical record on 142,973 patient-days during the study period. Of these 9,840 patients, 1 (0.01%) had home blood pressure documented in the structured flowsheets and 1,097 (11.1%) had home blood pressure recorded in the text of at least one of their provider notes. Of the 99,082 encounters with blood pressure documented in narrative notes, 2,060 (2.08%) included a home blood pressure reading in the text. Of the 142,973 patient-days with blood pressure documented in either narrative text or structured flowsheets, office blood pressure was recorded only in the structured flowsheets on 44,052 (30.8%) days, only in the notes on 52,869 (37.0%) days, in both sources on 45,751 (32.0%) days, and in neither source on 301 days (0.2%).

**Table 2:** Patient Characteristics

Variable	Value
Study patients, n	9,840

Age*, years (± SD)	62.2 (± 13.8)

Women, n (%)	5,495 (55.8)

Ethnicity	
White, n (%)	5,742 (58.3)

Black, n (%)	1,610 (16.4)

Hispanic, n (%)	1,564 (15.9)

Other, n (%)	924 (9.4)

English is the primary language, n (%)	8,036 (81.7)

Health insurance**	

Private, n (%)	3,332 (33.9)

Medicaid, n (%)	1,613 (16.4)

Medicare, n (%)	4,755 (48.3)

None, n (%)	140 (1.4)

Number of study encounters, n (± SD)	10.1 (± 8.7)

Median income by zip code, $ in tens of thousands (± SD)	49.8 (± 19.9)

Office systolic blood pressure***, mm Hg (± SD)	131.3 (± 18.6)

Office diastolic blood pressure***, mm Hg (± SD)	75.7 (± 7.2)

* Age at the time of each encounter, averaged over all encounters

** At the end of the study period

*** Average across all study encounters with documented office blood pressure using lowest documented office blood pressure from each encounter

Out of the 98,781 office blood pressure readings examined, 423 (0.43%) were reported as a range. However, of the 2,060 home blood pressure readings, 619 (30.0%) were reported as a range.

### Home vs. Office Blood Pressure Readings

**Figure 1:**
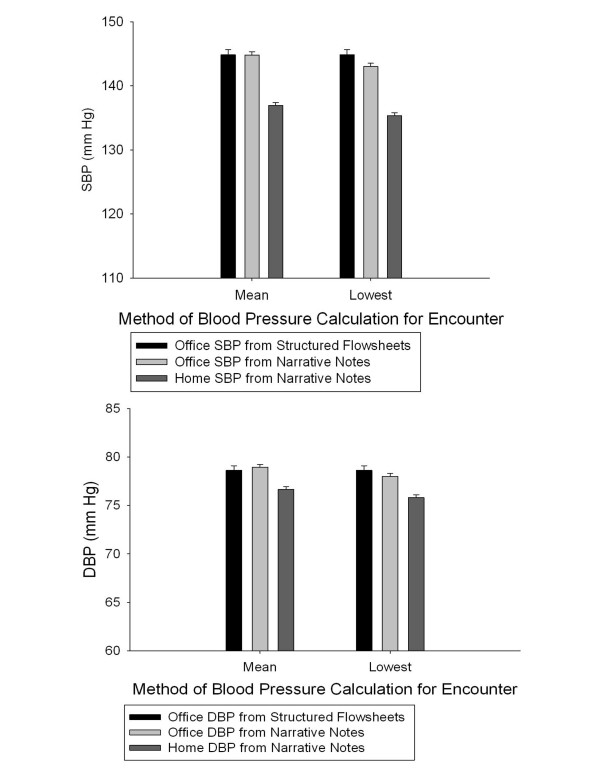
**Systolic Blood Pressure By Type of Blood Pressure Reading **Systolic Blood Pressure By Type of Blood Pressure Reading Office and home SBP readings from 2,060 encounters with a home blood pressure reading were compared. Wisps indicate standard error. b) Diastolic Blood Pressure By Type of Blood Pressure Reading.

Out of 1,759 days when both office and home blood pressure readings were documented, home blood pressure was lower on 1,059 (60.2%) days. On average, office blood pressure readings documented in structured flowsheets showed systolic blood pressure 9.6 mm Hg higher and diastolic blood pressure 2.5 mm Hg higher than home blood pressure readings (p < 0.0001 for both; Figure 1). Similarly, office blood pressure readings recorded in provider notes showed systolic blood pressure 7.5 mm Hg higher and diastolic blood pressure 2.1 mm Hg higher than home blood pressure readings (p < 0.0001 for both). In a multivariable analysis that adjusted for patient demographics and for clustering within individual patients, home systolic blood pressure was lower than office systolic blood pressure recorded in structured flowsheets and office notes by 9.4 mm Hg and 7.7 mm Hg, respectively (p < 0.0001 for both). Home diastolic blood pressure was lower than office diastolic blood pressure recorded in structured flowsheets and office notes by 2.5 mm Hg and 2.2 mm Hg, respectively (p < 0.0001 for both).

### Factors Influencing Home Blood Pressure Documentation

Frequency of reporting of home blood pressure varied widely between physicians. Of the 547 physicians who authored study notes, 304 authored more than 50 notes. Among these 304, the average physician recorded home blood pressure 2.05% of the time, ranging from 0% to 30.8% (interquartile range 0.4 to 2.8%). Only 6 (2.0%) of the 304 physicians recorded home blood pressures in more than 10% of the notes, while 62 (20.4%) physicians never recorded any.

**Figure 2:**
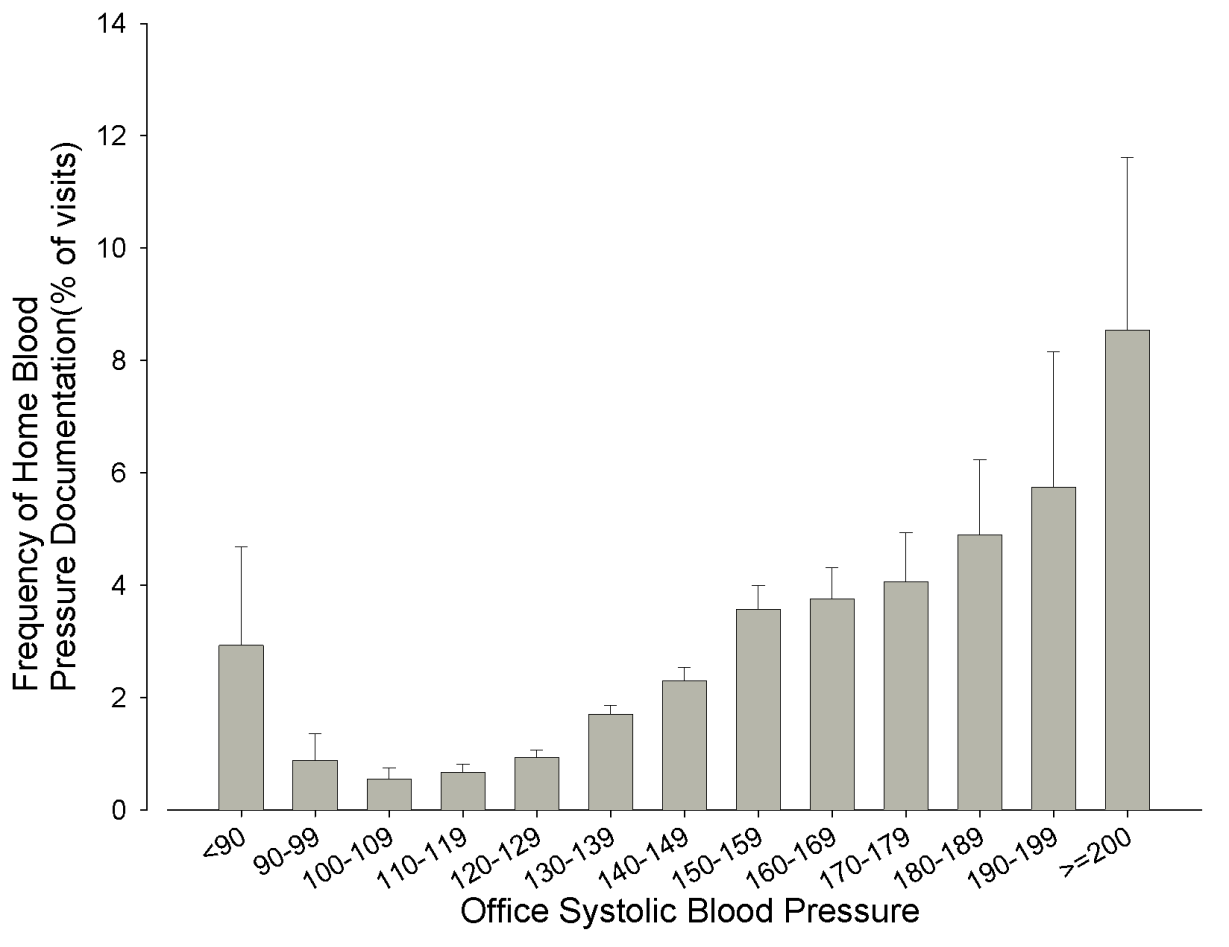
**Office Systolic Blood Pressure vs. Frequency of Home Blood Pressure Reporting **Office Systolic Blood Pressure vs. Frequency of Home Blood Pressure Reporting Frequency of documentation of home blood pressure in narrative notes was plotted against the lowest office systolic blood pressure from either narrative notes or structured flowsheets on the day of the encounter. Wisps indicate 95% confidence interval.

The frequency of home blood pressure documentation rose steadily with the lowest office blood pressure recorded during the encounter (Figure 2): from 0.5% for office systolic blood pressure between 100 and 109 mm Hg to 8.5% for office systolic blood pressure > 200 mm Hg. At the lower office blood pressure levels this relationship exhibited a J-curve character as the frequency of home blood pressure documentation rose to 2.9% for encounters with office blood pressure < 90 mm Hg.

In a multivariable analysis of 99,082 encounters (Table 3), the probability of home blood pressure being recorded increased by 23.0% for each 10 mm Hg increase in office systolic blood pressure (p = < 0.0001), by 6.2% for each $10,000 in median income by zip code (p < 0.0055), and by 17.7% for each decade in the patient's age (p = < 0.0001). The probability of home blood pressure documentation was lower by 53.9% for Hispanic patients relative to Caucasian patients (p = < 0.0001). There was no significant relationship between home blood pressure documentation and patient gender, office diastolic blood pressure, patient's insurance and primary language.

**Table 3:** Patient and Encounter Characteristics and Home Blood Pressure Documentation

Variable	Odds Ratio for Home Blood Pressure Documentation	p-value^1^
Female	1.081	0.30

Age^2^	1.177	**< 0.0001**

English not the primary language	0.871	0.37

Ethnicity^3^		

African-American	0.904	0.40

Hispanic	0.461	**< 0.0001**

Other	1.303	0.012

Health insurance^4^		

Medicare	0.909	0.32

Medicaid^5^	0.712	0.0218

None	1.412	0.26

Systolic blood pressure^6^	1.23	**< 0.0001**

Diastolic blood pressure^6^	1.049	0.1432

Median household income^7^	1.062	**0.0055**

^1^p-values for associations significant after Simes-Hochberg correction for multiple testing are boldfaced

^2^For every 10 years of age

^3^Compared to Caucasian

^4^Compared to private health insurance

^5^Includes FreeCare - a state-run health insurance program in Massachusetts that covers low-income families and individuals who do not qualify for Medicaid

^6^For every 10 mm Hg

^7^For every $10,000; based on median household income by zip code according to the 2000 census

## Discussion

In this retrospective study of over 140,000 encounters with over 9,800 patients, we found that home blood pressures are seldom documented in routine clinical care (only 2.08% of encounters that had any blood pressure recorded included a home blood pressure measurement). This relative scarcity of home blood pressure documentation may be partially attributable to a lack of reimbursement provided for the monitoring of home blood pressures, as our study implicates cost as a limiting factor in the use of home blood pressures. The limiting role of cost is shown through the finding that the likelihood of home blood pressure being documented increases 6.2% for every $10,000 increase in the median income of the patient's zip code. This lack of reimbursement is a situation which the American Heart Association(AHA), American Society of Hypertension (ASH), and Preventive Cardiovascular Nurses Association (PCNA) have highlighted as in need of remedy[29], and our study lends further support to this recommendation.

When home blood pressures were recorded, they were nearly always recorded in narrative notes rather than in the structured data. This difference is likely due to the extra time that it takes to record a blood pressure in the structured fields. Home blood pressure readings are typically reported by the patients directly to the providers whose time is more constrained than, for example, medical assistants, who enter the bulk of the structured blood pressure data. Faced with the pressure to make most efficient use of the 15-20 minute office visit, providers are more likely to include home blood pressure readings in the narrative of the encounter note but not take the time to also enter it into the appropriate structured field. As a result, home blood pressure readings recorded in narrative but not structured data are not easily accessible to other providers and are not available for quality of care monitoring and/or research. Improvement of reimbursement of healthcare providers for supporting, discussing and documenting home blood pressure monitoring (in addition to office blood pressure measurement) could potentially increase home blood pressure documentation overall and particularly in structured format. A compensated practice change that would include health care team providing education and self management support for patients to monitor their blood pressure at home, as well as coaching and regular discussions about and documentation of home blood pressures between patients and the health care team, would be an optimal approach.

Data collected in the course of routine clinical care, and particularly data, such as home blood pressure readings, that are collected irregularly, could be subject to selection bias. It was therefore important to validate our findings to establish whether home blood pressure records in routine clinical care provided an adequate representation of reality. To this end we performed a quantitative comparison of home and office (which are recorded less selectively) blood pressure readings. Compared to the office blood pressure readings from structured data, home systolic blood pressures were 9.6 mm Hg lower and home diastolic blood pressures were 2.5 mm Hg lower, consistent with the findings of several prospective studies[30]. This result provides external validation for the home blood pressure readings recorded in routine patient care, suggesting that despite the likely selection bias they provide an accurate representation of the patients' blood pressure. While the average home blood pressure measurements were significantly lower than the average office measurements, only 60% of individual home blood pressure readings were lower than office measurements. This finding is likely due to variability in home and particularly office blood pressure measurements, and is broadly consistent with previously reported results of prospective studies[31]. The difference in home and office blood pressures may be due to white coat hypertension, a documented phenomenon where the clinic setting and/or the presence of a physician can raise a patient's blood pressure[32]. In the case of our study, home blood pressures may also be depressed as compared to office blood pressures because the physician may be more likely to ask the patient about their home blood pressure measurements if their office blood pressure is unusually high on a particular day. It is also possible that the patients may be reporting lower home blood pressure readings in order to please their physicians or to avoid having their medications increased (due to the cost of medications or fear of side effects). However, this last reason is less likely to be a significant contributing factor in our data because it would be expected to lead to larger differences between office and home blood pressure than those observed in prospective studies; this was not observed.

Our study found that higher office systolic blood pressure, the patient's income as measured by median income of the patient's zip code, and older age were significantly associated with an increased probability of documentation of home blood pressure. For every 10 mm increase in office systolic blood pressure, the probability of home blood pressure being recorded rose by 23.0%. One explanation for this finding may be that physicians are unlikely to inquire about and/or record home blood pressure when office blood pressure is normal -- recording home blood pressure is usually done to justify treatment decisions that would have been different if only office blood pressure were available. For example, the physician may request home blood pressure readings to rule out white coat hypertension or to confirm that an unusually elevated blood pressure is inconsistent with the patient's long-term trend. Our study also showed a 6.2% increase in documentation of home blood pressure for every $10,000 increase in the median income of the patient's zip code. This finding could in part be attributed to an increased ability of individuals with higher income to afford a home blood pressure monitoring device and further supports the call by the AHA, ASH and PCNA for the reimbursement of home blood pressure monitoring costs, as it implies that the cost may be a key factor in determining whether a patient is able to monitor their blood pressure at home[29]. We also found a 17.7% increase in the probability of the patient's home blood pressure being documented for each additional decade of the patient's age. This could be due to the known phenomenon of greater adherence of elderly patients to physician's recommendations, leading to an increased likelihood that elderly patients will actually take home blood pressures and report them to the physician[33]. It is also possible that older/retired patients may simply have more time to perform home blood pressure measurements. Hispanic patients were less likely (by 55%) than Caucasian patients to have home blood pressure documented, independent of the primary language and household income; difference between other ethnic groups have not reached statistical significance. Further research is needed into possible treatment disparities reflected in this finding.

Our investigation has several limitations. It was restricted in scope to diabetic hypertensive patients of physicians affiliated with two academic hospitals in Eastern Massachusetts; this could limit its generalizability. The data analyzed in our study was collected between 5 and 10 years ago and the patterns of documentation of home blood pressure measurements could have changed since that time. We have not conducted an analysis of the temporal order of factors that affect documentation of home blood pressure as precise temporal information is frequently not available in narrative documents. Due to the retrospective nature of our analysis, the data were collected for the purpose of routine care rather than for the analysis, and some of the data were missing. If the missing data were not missing at random with respect to the outcomes, our findings could be biased. However, our results were broadly consistent with previously reported prospective studies, supporting our conclusions. Most of the home blood pressure data in our study was computationally abstracted from narrative provider notes in the EMR. Narrative documents may not always contain information sufficient to make an unequivocal assessment of the location and/or timing of blood pressure measurement, as reflected in the disagreement between manual ratings of blood pressure readings in our study. However, overall our findings were congruent with previously published results of prospective studies, providing external validation to the technology.

## Conclusions

Home blood pressure readings in the documentation of routine clinical care provide a valid representation of the patient's condition and could be used in the care of individual patients, quality assurance and research. However, their documentation remains sporadic, and they are primarily recorded in narrative documents rather than structured data. Furthermore, our study suggests that the cost of home blood pressure monitoring may be a significant factor influencing the prevalence of its use, giving further support to the need for reimbursement of home blood pressure monitoring costs.

## Competing interests

The authors declare that they have no competing interests.

## Authors' contributions

MHK, MS and AT designed the study. MHK, EB, and KB collected the data. MHK, MS and AT conducted data analysis. MHK drafted the manuscript. MHK, EB, MS, KB, JSE, and AT critically revised the manuscript for important intellectual content. All authors read and approved the final manuscript.

## Pre-publication history

The pre-publication history for this paper can be accessed here:

http://www.biomedcentral.com/1472-6963/10/139/prepub
